# The risks perceived by the consumer in the acceptance of electronic commerce. A study of Bolivia

**DOI:** 10.1371/journal.pone.0276853

**Published:** 2022-11-28

**Authors:** Orly Carvache-Franco, Jose Loaiza-Torres, Carolina Soto-Montenegro, Mauricio Carvache-Franco, Wilmer Carvache-Franco

**Affiliations:** 1 Universidad Católica de Santiago de Guayaquil, Facultad de Economía y Empresa, Guayaquil, Ecuador; 2 Universidad Católica Boliviana “San Pablo” Sede Tarija, Tarija, Bolivia; 3 Universidad Espíritu Santo, Samborondón, Ecuador; 4 Escuela Superior Politécnica del Litoral, ESPOL. Facultad de Ciencias Sociales y Humanísticas, Guayaquil, Ecuador; Asia University, TAIWAN

## Abstract

The risks perceived by consumers in online shopping can negatively affect the acceptance of electronic commerce, however, the perceived risks are a cultural aspect that can affect consumers differently in different countries. This research examines what dimensions of perceived risk affect the acceptance of electronic commerce in Bolivia, a developing country. The design is quantitative, the TAM model is used with the variables: risk of misuse of information, risk of not obtaining the benefits of the product and risk of inefficiency of the functionality. The results show that the three risks examined do not affect the acceptance of electronic commerce, and that the variables perceived usefulness and ease of use have a positive effect on the acceptance of electronic commerce. The theoretical implications of this study provide empirical evidence from Bolivia, which shows strong variables of perceived usefulness and ease of use, which mitigates the effect of risk´s perception by the consumer, the results are explained in the context of the COVID-19 pandemic that accelerated the rapid acceptance of electronic commerce, increasing the perception of usefulness and ease of use of online shopping. As practical implications, this research provides managers and administrators of online businesses with knowledge about the effect of risk variables perceived by consumers.

## 1. Introduction

Websites are the channels used by companies to attract customers around the world [1]. In many developing countries consumers had online shopping options [2]. However, this situation changed drastically with the Covid_19 pandemic, causing an increase in the implementation of online stores and an increase in sales due to consumer demand [3–5].

Electronic commerce encourages companies to expand the market and attract new customers, due to the advantages such as shorter delivery times and lower costs of products and services [6]. However, despite the advantages of electronic commerce, consumers perceive a greater risk in online purchases than in physical purchases [7].

The influence of perceived risk on the consumer is an important factor in the entire process of electronic commerce [8]. In the literature, various variables of perceived risk have been studied. There is evidence that functionality inefficiency risk, information misuse risk, and failure to gain product benefits risk negatively affect online shopping [9–11].

The risk of misuse of information in electronic commerce is a privacy risk that involves the release of credit card information or financial information [12], customers perceive the risk as the potential action of the company that shares their data with third parties without their consent [13], this increases the risk for customers who submit credit card information and influence their purchase intention online [14].

The risk of not obtaining the benefits of the product in e-commerce it refers to the risk that the product received does not meet the expected benefits when making the purchase online [15], the customers perceives this risk since there is no direct experience with the product in electronic commerce [15, 16]. This risk is also perceived by customers when they have higher expectations about product features or performance [9, 17].

In e-commerce, customers have to perform functions like find, choose, order, pay, receive, return, etc. on the websites, so they perceive the risk of inefficiency of the functionality due to the possibility of doing something improper on the seller’s website that harms them in costs or quality of the product to be received [11, 18].

The objective of this research was to examine the acceptance of electronic commerce by customers in Bolivia, using the TAM model integrated with three risk variables for consumers: (1) risk of misuse of customer information, (2) risk of not obtaining the benefits of the purchased product and (3) risk of inefficiency in the functionality. Thus, this study aimed contributes to the gap in the literature on what dimensions of perceived risk affect e-commerce uptake by presenting evidence from Bolivia a developing country where consumer behavior towards risks may be different because the culture of the country can affect the perceived risk of customers’ online purchase intention [4, 19] and because there is evidence that these risk variables negatively affect the acceptance of electronic commerce [9–11] and evidence that does not affect the acceptance of electronic commerce [20].

## 2. Literature review

### 2.1. TAM model and the acceptance of electronic commerce

Some models have been used to examine the acceptance of technology, such as the reasoned action model (TRA) [21, 22], the theory of planned behavior (TPB) [23] and the technology acceptance model (TAM) that establishes that perceived usefulness and ease of use lead to the acceptance of technology [24], model to which other constructs such as perceived enjoyment and emotions have been incorporated [25].

Applying the TAM model to customer acceptance of e-commerce, the perceived ease of use variable refers to the use and operation of the merchant’s e-commerce website, whereas the perceived usefulness variable is related to customer preferences and the benefit they receive, such as the speed in the purchase and delivery of services [26]. Several studies have found a positive relationship between perceived usefulness and acceptance of e-commerce [9, 10, 27–30] and ease of use and acceptance. of the e-commerce customer [9, 10, 28, 31, 32]. Park et al. [28] established a relationship between the two variables and the acceptance of electronic commerce in the USA and Korea; however, they discovered cultural differences between consumers in the two countries.

### 2.2. The perceived risks in accepting electronic commerce

With the digital transformation, the behavior of the online consumer has been studied in its relationship with social media marketing [33] as well as the satisfaction of the online user with the main resources used such as software [34], which is related to utility and satisfaction [35] for which the data, the operation of the software and the results of the software [36] that provide utility to users are important users [37].

When consumers encounter unwanted situations during shopping, they perceive different types of risks, and the higher the perceived risk, the less likely they are to make the purchase [38]. The risks related to shopping have been studied based on the theory of risks in consumer behavior of Cox [39, 40] and Bauer [41].

Various risks perceived by the consumer have been studied such as: quality risk, the possibility that the product does not work as designed [42], risk of potential loss of time related to making a bad purchase decision [42], risk in delivery or potential loss due to receiving incorrect products [43, 44], risks associated with warranty issues and product problems [43, 44], privacy risk due to possible loss of information or misuse of customer information [44–46], social risk, the potential loss of social status from purchasing an unpopular product [42], and financial risk, the possible loss of money associated with the initial cost of the product plus the cost of maintenance, and the possible loss due to fraud [42].

The literature shows empirical evidence of the effect of the perceived risks on electronic commerce. For example, Featherman and Pavlou [45, 46] examined the dimensions of performance, privacy, financial, and time risks and found that the perceived risk negatively correlates with customer acceptance of electronic commerce. Ko et al. [19] examined the risks perceived in online purchases in Korea and the USA. They identified that the risk is greater for inexperienced or less experienced users and that in both countries, the perceived risk is related to the acceptance of electronic commerce. They also established risk as a critical factor and unveiled cultural differences regarding consumers’ perceptions of risk in both countries. Moreover, Park et al. [28] determined that consumers’ perceived risk in online purchases in the USA and Korea directly impacts their internet purchasing behavior.

Barnes et al. [47] found that consumers from France, Germany, and the USA perceive risk in online shopping, creating uncertainty in their purchase decision and concern about the costs of what is purchased. In contrast, in India, Arora and Rahul [48] established that security, privacy, product, and delivery risks in e-commerce acceptance do not affect customer e-commerce acceptance. Chen et al. [12] examined the perceived risk in three dimensions: uncertainty of transaction cost, product performance, and individual consumer anxiety. Their findings indicated that these dimensions have different effects on the purchase intention of Chinese customers.

Alraja and Aref [9] found that the risk of information misuse, the risk of not benefiting from the product, and the risk of inefficient functionality are negatively related to e-commerce acceptance. Similarly, Glover and Benbasat [11] analyzed the risk of information misuse, the risk of not benefiting from the product, and the risk of inefficient functionality and its effect on online shopping.

The perceived risks in electronic commerce affect consumer reliability [17]. For this reason, social influence is an essential factor in consumer acceptance of electronic commerce [49, 50], which cause them uncertainty or concern about their decision to buy online [19, 47]. Among the main risks that consumers perceive in online purchases that have been identified in the literature are the risk of security and privacy [20, 48, 51, 52]. When the risks perceived by consumers during online purchases are strong, they mitigate the effect of the variables ease of use and perceived usefulness of the product [53, 54].

Some authors have claimed that privacy risks, product benefit risks, and functionality risks affect consumer purchasing decisions [9, 10]. However, these risks do not always affect online purchase acceptance decisions [20] so it is not a generalized theory that these risks of misuse of information, failure to obtain benefits from the product and inefficiency in functionality affect the online purchase decision of consumers. The culture of the country can affect the perceived risk of customers’ online purchase intention [4, 19]. Therefore, there is a gap in the literature to know if the risks perceived by consumers affect online purchases in other contexts such as developing countries that show differences in the implementations of electronic commerce with developed countries where there is generally evidence of the risks perceived by consumers in online shopping.

This research examined the technology acceptance model (TAM), integrating the perceived risk in three dimensions: information misuse risk, failure to gain product benefits risk, and functionality inefficiency risk in Bolivia a developing country, to verify if these variables were related to the acceptance of electronic commerce.

**Fig 1:**
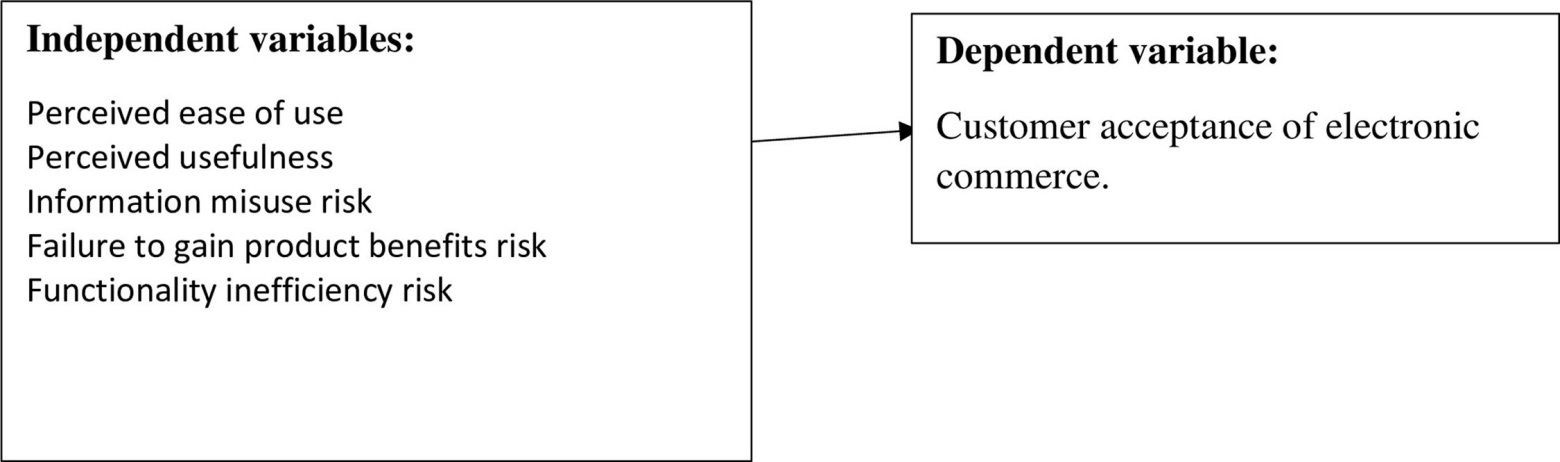
Relationship between the variables.

Fig 1 shows the research model and the variables identified in the literature review.

Because the perceived ease of use, including the use and operation of e-commerce websites, is a predictor of customer acceptance of electronic commerce [9, 10, 28, 31, 32], we propose the following hypothesis.

H1 = Perceived ease of use has a positive relationship with customer acceptance of electronic commerce.

Since perceived usefulness, including improvements in costs, speed of purchase, and delivery services, is a predictor of the acceptance of electronic commerce [9, 10, 27–29], we formulate the following hypothesis.

H2 = Perceived usefulness has a positive relationship with customer acceptance of electronic commerce.

Because security risks, such as the misuse of customer information by the business, can affect e-commerce adoption by restricting people from making online purchases [9, 14, 55], we posit the following hypothesis:

H3 = The perceived risk of misuse of information has a negative relationship with customer acceptance of electronic commerce.

Since the risk of purchasing a product through an online store and not obtaining the anticipated benefits or fulfilling customers’ expectations can affect the adoption of electronic commerce [9, 56, 57], we propose the following hypothesis:

H4 = The perceived risk of failure to gain product benefits has a negative relationship with customer acceptance of electronic commerce.

Because there is a functionality risk when customers are using e-commerce, either finding, choosing, ordering, paying, receiving, or returning their order, which could restrict them from making online purchases [11, 18], we formulate the following hypothesis.

H5 = The perceived risk of functionality inefficiency has a negative relationship with customer acceptance of electronic commerce.

## 3. Methodology

The design is quantitative, transversal, not experimental. The sample was composed of 395 surveys collected in Bolivia from electronic commerce consumers. According to Byrne’s [58] criteria, the number of surveys for each question is 8 in structural equations. Considering that there are 29 questions, this gives 232 necessary surveys, so the 395 surveys obtained exceeded that amount.

A validated instrument based on previous studies was used as the survey [9, 11, 46, 59] with 29 questions for the constructs: perceived ease of use, perceived usefulness, perceived risk in information misuse, perceived risk in failure to gain product benefits, and perceived risk in functional inefficiency. The questions were measured with a 5-point Likert scale. The sampling method used was non-probabilistic for convenience. The surveys were administered online to the e-commerce consumers with availability to answer the questions. The Project was approved by the ESPOL University Research Dean with Code FCSH-14-2021 and the consent of the participants was informed in writing at the beginning of the questionnaire where the participants agreed to participate in the study.

### 3.1. Reliability analysis

Based on confirmatory factor analysis (CFA), the convergent validity, discriminant validity, and reliability of the scales were measured. Reliability was assessed based on Composite Reliability (CR) by the degree to which items are free from error and can produce consistent results. The recommended appropriate CR value is ≥ 0.70 [60, 61]. Convergent validity was verified using factor loadings greater than 0.5 and a minimum average variance extracted (AVE) of 0.5 [60]. The discriminant validity was evaluated through a minimum AVE value of 0.5 [60].

### 3.2. Structural model

A structural model was used, which is a multivariate technique to analyze causal relationships, SEM is a useful method as it allows one to estimate a set of separate, interdependent multiple regression equations simultaneously, in a specific structural model [62]. Therefore, SEM is the most suitable analysis to estimate the strength of the casual relationship of these constructs used.

It was used a set of indices to examine the structural model [63] as the Comparative Fit Index (CFI), which compares the fit of a target model to the fit of an independent model [64, 65]; the Goodness of Fit Index (GFI), which measures the fit of a model when compared to another model [62]; the Normed Fit Index (NFI), which assesses the model by comparing the X^2^ value to the X^2^ of the null model [62]. The values of CFI, GFI, Incremental Fit Index (IFI), and NFI are recommended to be > 0.9 [66]. In addition, the Root Mean Square Error of Approximation (RMSEA) was measured [66, 67]. Its values must be between 0.05 and 0.08 to be acceptable [68].

The model-fit indices are expected to exceed the respective common acceptance levels suggested by previous authors, demonstrating that the model exhibited a good fit with the data collected. Once the fit indices have been demonstrated, the next step is to examine the path coefficients of the structural model.

### 3.3 Path analysis

The structural model reflecting the assumed linear, causal relationships among the constructs. The test of structural model was performed using the following steps: (1) estimating the path coefficients, or the strengths of the relationships between the dependent variables and independent variables, and (2) estimate the R-square value which is the amount of explained variance of the independent variables. SPSS AMOS software will be used to determine The path coefficients in the SEM model obtaining standardized path coefficients (β), standard error, p-value and hypotheses result. The hypotheses will be supported or accepted for values of p-value with a level of significance of 0.05

## 4. Results

Descriptive results were obtained on the population under study, which are shown in Table 1.

**Table 1:** Descriptive results.

Socio-demographic variable	Frequence	Percent (%)
**Gender**	395	100
Male	179	45.3
Female	216	54,7
**Age**		
18–29 years old	249	63.0
30–55 years old	146	37.0
**Level of education**	395	100
Bachelor’s degree	262	66.33
University´s degree	125	31.64
Master’s degree	8	2.03
**Departament**	395	100
Beni	3	0.8
Chuquisaca	8	2.0
Cochabamba	29	7.3
La Paz	60	15.2
Oruro	2	.5
Pando	3	.8
Potosi	12	3.0
Santa Cruz	40	10.1
Tarija	238	60.3

Table 1 shows the descriptive results of the survey.

Reliability was evaluated through CR (composite reliability), which measures internal consistency in scale items. The CR measure obtained in this study was between 0.6913 and 0.832, which are between the recommended threshold of ≥ 0.7 [60, 61], except for the Functionality Inefficiency Risk construct, which CR was 0.547.

Convergent validity was assessed through factor loadings and the average variance extracted (AVE). It was observed that all factor loadings (Standardized factor Loadings) were greater than 0.508, exceeding the threshold agreement of ≥ 0.5 [60], and the variance extracted AVE was greater than 0.59, also exceeding the recommended minimum of not less than 0.5 [60]. The discriminant validity was evaluated through the variance extracted AVE with a value greater than 0.59, higher than the recommended 0.5 [60].

Table 2 shows the standardized factor loadings (Standardized factor Loadings) for each construct, CR (composite reliability), and AVE (average variance extracted).

**Table 2:** Reliability and factor loadings.

	Constructs /Measurement Items	Standardized factor Loadings	CR	AVE
**Information Misuse Risk**			0.7015	0.68
Np3	The seller on the website may not be a real trader.	0.604		
Np4	My financial information may be hacked by another party and misused.	0.732		
Np6	The online seller may not have sufficient firewalls to protect my personal information from hackers.	0.710		
Np7	My personal information may be used to send disturbing e-mails without my consent.	0.665		
**Failure to Gain Product Benefits Risk**			0.6913	0.73
Np8	The product characteristics and models that are displayed via the website might not be real.	0.783		
Np9	Maybe I cannot identify the features of the product that I want to buy.	0.699		
Np10	Maybe the product that was previewed on the website differs from the product that is shipped.	0.758		
Np11	Maybe I do not get the product at the expected time or when I need it.	0.677		
**Functionality Inefficiency Risk**			0.547	0.59
Np12	Maybe I cannot efficiently use the website and its tools.	0.508		
Np14	Maybe the online seller makes me pay additional costs such as shipping, taxes, and customs duties.	0.617		
Np16	The online seller may add incorrect information about the warranty and maintenance.	0.644		
**Perceived ease of use**			0.791	0,67
Np22	Online shopping increases my ability to make a purchase decision.	0.660		
Np23	Online shopping speeds up the process of getting what I need.	0.663		
Np24	Online shopping enhances the effectiveness of the purchase process.	0.730		
Np25	Online shopping improves my purchasing performance.	0.729		
**Perceived Usefulness**			0.832	0,68
Np17	It is easy for me to purchase over the internet.	0.569		
Np18	I think it’s easy to gain e-purchasing skills.	0.564		
Np19	It’s easy to learn how to make purchases through the internet.	0.723		
Np20	E-purchasing is understandable and clear.	0.755		
Np21	When I make e-purchases, it is easy to use all the website tools.	0.796		
**E-commerce acceptance**			0.700	0,61
Np29	I think using a website to buy gives me more options.	0.650		
Np28	I frequently use a website to buy many things that I need.	0.573		
Np27	I intend to use a commercial website to purchase if given a chance.	0.666		
Np26	In the future, I think I should use a website to shop if given a chance.	0.543		

### 4.1 The structural model

The χ2/df ratio obtained was 1.9 which is lower than 3 the benchmark [68]. The fit of the model was analyzed through several indicators: the Comparative Fit Index (CFI) obtained was 0.940, the Goodness of Fit Index (GFI) was 0.913, and the Incremental Fit Index (IFI) was 0.94.1, which satisfy the recommended values of 0.9 for CFI, GFI and IFI [65]. Other indices analyzed were the Normed Fit Index (NFI) which was 0.883, and the Root Mean Square Error of Approximation (RMSEA), with 0.057, both acceptable since the recommended values were 0.05 and 0.08 [68]. Table 3 shows the values of the indices as they met the thresholds recommended in the literature. Therefore, for the overall measurement, it is concluded that the model exhibited a good fit with the data examined.

**Table 3:** Results of the fitting model.

Fit Indices	Benchmark	Value
**Absolute fit measures**		
CMIN (χ 2)		455.90
DF		240
CMIN (χ 2)/DF	3	1.90
GFI (Goodness of Fit Index)	0.9	0.913
RMSEA (Root Mean Square Error of Approximation)	0.08	0.057
**Incremental fit measures**		
AGFI (Adjusted Goodness of Fit Index)	0.80	0.891
NFI (Normed Fit Index)	0.90	0.883
CFI (Comparative Fit Index)	0.90	0.940
IFI (Incremental Fit Index)	0.90	0.941
RFI (Relative Fit Index)	0.90	0.866
**Parsimonious fit measures**		
PCFI (Parsimonious Comparative of Fit Index)	0.50	0.818
PNFI (Parsimonious Normed Fit Index)	0.50	0.768

### 4.2. Path analysis

**Fig 2:**
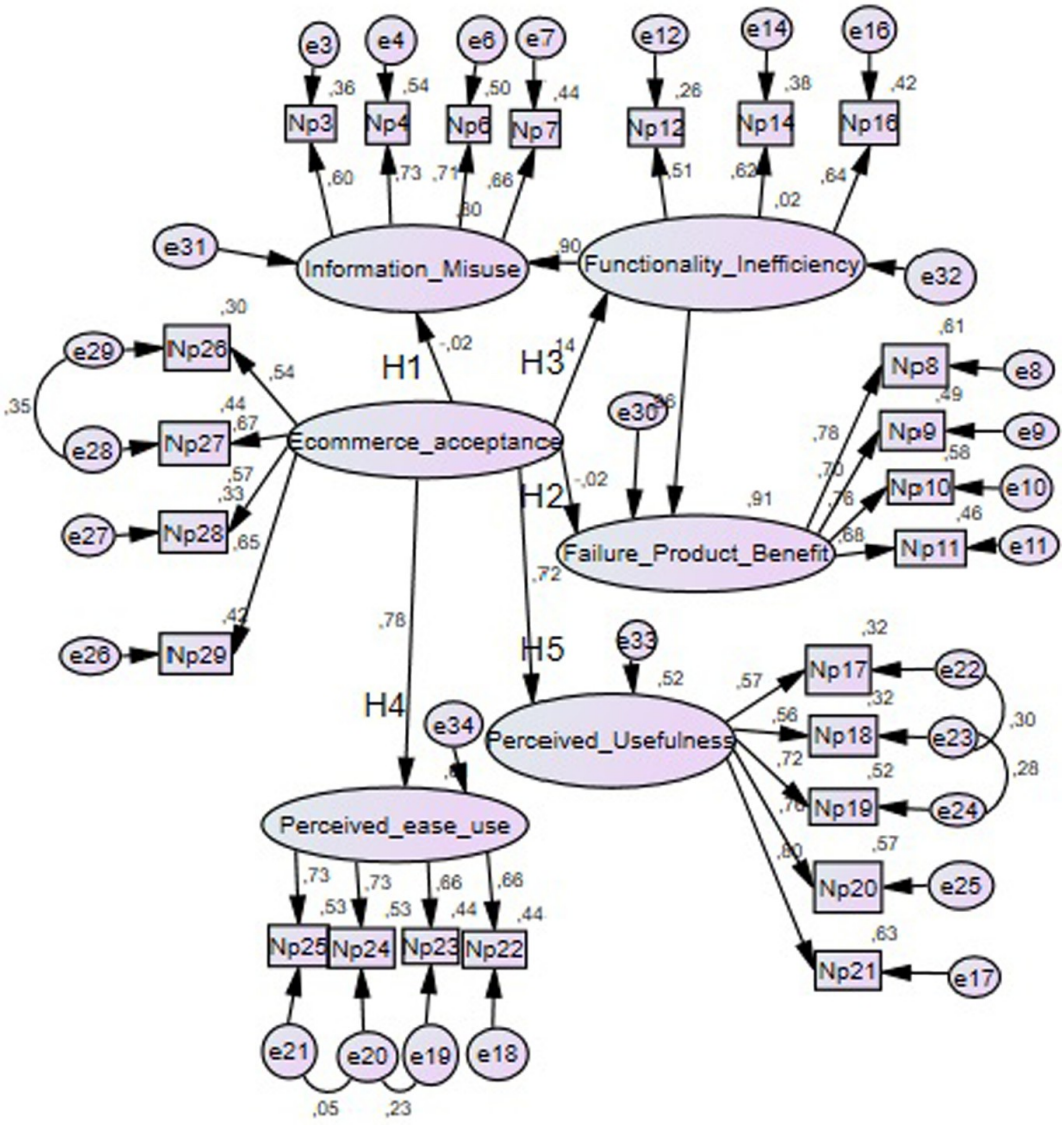
Structural equation model. Source: Authors.

The relationship between the independent and dependent variables was examined through a path analysis. The structural model test was performed using SEM, and the coefficients (β) and R2 were estimated, obtaining a value of 0.51, which is considered moderate [69] and indicates that the variation of the dependent variable is explained by 51% of the variation of the independent variables (Fig 2).

Table 4 shows the results of the hypothesis testing. The variable Perceived Usefulness had a β = 0.752 and a level of significance P = 0.00, so hypothesis H1 is supported. The variable Perceived ease of use had a β = 0.843 and a level of significance P = 0.00; therefore, hypothesis H2 is supported. The Information Misuse Risk variable had a β = -0.018 and a level of significance P = 0.769, so hypothesis H3 is not supported. The Failure to Gain Product Benefits variable had a β = -0.036 and a significance level P = 0.639; therefore, hypothesis H4 is not supported. Finally, the Functionality Inefficiency Risk variable had a β = 0.120 and a significance level P = 0.058, so hypothesis H5 is not supported.

**Table 4:** Results of hypothesis testing.

Path	β	C.E.	C.R.	P	Result
(H1) Perceived Usefulness <--- E-commerce acceptance	0.752	0.094	7.970	0.00	Supported
(H2) Perceived ease of use <--- E-commerce acceptance	0.843	0.091	9.293	0.00	Supported
(H3) Information Misuse Risk <--- E-commerce acceptance	-0.018	0.061	-0.293	0.769	Not Supported
(H4) Failure to Gain Product Benefits Risk <--- E-commerce acceptance	-0.036	0.076	-0.469	0.639	Not Supported
(H5) Functionality Inefficiency Risk <-- E-commerce acceptance	0.120	0.064	1.894	0.058	Not Supported

## 5. Discussion

The objective of this research was to examine customer acceptance of e-commerce using the TAM model. The variables of perceived ease of use and perceived usefulness integrated with the variables of perceived risk: misuse of information, the impossibility of obtaining benefits from the product and the inefficiency of functionality, were studied to contribute to the gap in the literature to determine what risks affect the acceptance of electronic commerce by customers.

The results show that perceived usefulness and ease of use are positively related to the acceptance of electronic commerce. These findings are consistent with the results found by Alraja and Aref [9] in Omani and Lim and Ting [10] in Malaysia. Other scholars such as Yan et al. [32] and Shukla and Sharma [31], also determined that perceived ease of use positively affects the acceptance of electronic commerce. Moreover, in Mexico, Ventre and Kolbe [29] found that perceived usefulness is positively related to customer acceptance of e-commerce.

The positive relationship of the variables of the TAM model, perceived usefulness and ease of use, with the acceptance of electronic commerce, can be explained by the social confinement experienced during the COVID-19 pandemic, in which consumers resorted to online shopping as a way to cope with the mobility restrictions. In Bolivia, e-commerce significantly developed since customers perceived greater usefulness in online shopping, such as cost advantages and the convenience of home delivery. They also experienced the ease of use of the website by being able to search for products or choose them from the catalog. As a consequence, both perceived usefulness and ease of use became predictors of customer e-commerce acceptance.

Regarding information misuse risk, failure to gain product benefits risk, and functionality inefficiency risk, the results show that none of these risks are related to customer acceptance of electronic commerce. These findings are similar to those obtained by Arora and Rahul [48] in India, who found that the security risk, privacy risk, and product risk do not affect customer acceptance of electronic commerce.

These results differ from those reported by other academics, who found that functionality inefficiency risk, information misuse risk, and failure to gain product benefits risk negatively affect online shopping [9, 10]. On the other hand, other studies have identified different risk variables and their incidence on customer acceptance of online purchases. For example, Chen et al. [12] found product performance risk; Featherman [45] and Pavlou [46] established privacy risks; Barnes et al. [47], and Park et al. [28] described the risk and uncertainty in what is purchased online; Thakur and Srivastava [38] identified privacy and security risks; finally, Habib and Hamadneh [4] reported the existence of risk in online transactions but also the increase in confidence during the COVID-19 pandemic.

The culture of the country can affect the perceived risk of customers’ online purchase intention [4, 19]. The lack of relationship between perceived risk and online purchases in Bolivia can be explained from a cultural perspective. Bolivia, a developing country with little experience with electronic commerce before the COVID-19 pandemic, experienced a rapid and strong increase in online shopping and a quick acceptance of e-commerce during the pandemic. In light of this, businesses implemented modern e-commerce platforms to increase consumers’ trust and reduce risk perceptions. For example, the functionality inefficiency risk has been reduced since the introduction of open source e-commerce platforms that generate confidence in many companies and the use of gateways or payment methods such as PayPal or Google wallet, 2Checkout, EBANX, Payme, Pagonet, BaniPay, Khipu, which have minimized the security risk of payment transactions.

The perceived risk of information misuse has been reduced since Bolivian users can choose between cash and credit card payments and are not required to release a lot of personal information; also, the websites now mention the businesses’ privacy policies. The risk of failure to gain product benefits has been mitigated by including information on the dimensions, materials, and other product characteristics and the trader’s return policies.

This study sheds light on the topic of perceived risk in Bolivia. The results show that functionality inefficiency risk, information misuse risk, and failure to gain product benefits risk are not related to customer acceptance of electronic commerce. However, Bolivian customers have found substantial advantages in the ease of use of e-commerce platforms and have a high perception of the usefulness of online purchases during the COVID-19 pandemic. Combined with the sense of security offered by the current electronic commerce platforms, these variables have allowed a wide acceptance of online purchases in this developing country.

This study has theoretical implications since it shows that the strong variables of ease of use and perceived usefulness mitigate the effect of the variables of perceived risk in consumers in online purchases, and these variables do not affect the acceptance of electronic commerce. The perceived usefulness and ease of use of online shopping are the predictors of the acceptance of electronic commerce in Bolivia due to the fact that the COVID-19 pandemic favored the adoption of online shopping. The previous literature mentions that high perception of risk in the consumer, mitigates the effect of ease of use and perceived usefulness variables [53, 54], in this study the opposite effect is found, that is, that the perception of ease of use and perceived usefulness of the consumer is high due to the impulse to buy online that was generated during the covid_19 pandemic, which mitigated the effect of the perception of risks perceived by consumers.

According to the theory of risks in consumer behavior, the greater the amount of risk perceived by the consumer, the less possibility of purchasing [39–41]. Evidence of this has been found in contexts where the risks affect consumer acceptance of electronic commerce. However, this study shows that the culture of the country also affects their perception of security in e-commerce platforms and the perception of risks for online purchases, contributing theoretically to the existing literature.

As practical implications, this study provides managers and administrators of online businesses with knowledge about the risk perception of online buyers, to help them plan communication actions and plans to improve websites to minimize buyers’ perception of risk and increase the acceptance of electronic commerce.

## 6. Conclusions

This research examined customer acceptance of e-commerce using the TAM model by integrating perceived ease of use and perceived usefulness with perceived consumer risks of: risk of misuse of information, risk of not obtaining the benefits of the product and risk of inefficiency of the functionality, using data from Bolivia, a developing country.

The results indicate that the perceived ease of use and the perceived usefulness are positively related to the acceptance of electronic commerce by the client, but no relationship was found between the risk variables: risk of misuse of information, the risk of not obtaining the benefits of the product and the risk of inefficiency of the functionality, with the acceptance of electronic commerce by the customers.

As theoretical implications, this study provides evidence of perceived risks in the context of Bolivia, a developing country, which shows that the existence of strong variables of perceived usefulness and ease of use, which mitigates the effect of risk´s perception of misuse of information, risk of not obtaining the benefits of the product, and risk of inefficiency of the functionality, and that these risk variables do not negatively affect the acceptance of electronic commerce by the consumer, which is explained in the context of the pandemic covid_19 and the increase in online shopping. Therefore, this research contributes to the existing gap in the literature on the dimensions of risk perceived by the consumer that affect the acceptance of electronic commerce.

This study has practical implications as it offers knowledge to managers and administrators of online businesses about the perception of risk in online purchases by consumers in developing countries, which allows them plan communication actions and plans to improve websites to minimize buyers’ perception of risk and increase the acceptance of electronic commerce.

The main limitation of this study was the temporality of the data, which was collected during the COVID-19 pandemic. Therefore, future studies are suggested in other developing countries and during the post covid_19 pandemic to confirm the results and incorporate other variables of customer behavior in the perception of risk of online purchases.

## Supporting information

S1 Data SPSS database of the analyzed data is attached. (SAV)
